# Modified Valsalva test differentiates primary from secondary cough headache

**DOI:** 10.1186/1129-2377-14-91

**Published:** 2013-11-19

**Authors:** Enrico Ferrante

**Affiliations:** 1 Neurosciences Department, Headache Centre, Ospedale Niguarda Cà Granda, Piazza Ospedale Maggiore 3, 20162 Milano, Italy

**Keywords:** Cough headache, Valsalva manoeuvre, Chiari malformation, Spontaneous intracranial hypotension

## Abstract

I suppose that the patient number 14, reported in the article of RJ Lane et al. on “Modified Valsalva test differentiates primary from secondary cough headache ”in a recent issue of your esteemed journal, was probably suffering from spontaneous intracranial hypotension syndrome (SIH) caused by cervical manipulation.

## 

Sir,

I have read with interest the article of RJ Lane et al. on “Modified Valsalva test differentiates primary from secondary cough headache ” [1] in a recent issue of your esteemed journal. “They examined 16 consecutive cough headache patients using a modified Valsalva test (exhalation into the connecting tube of a standard anaeroid sphygmomanometer to 60 mm Hg for 10 seconds).

A positive response was recorded if the manoeuvre provoked headache. All patients subsequently underwent brain MRI. None of the patients had neurological signs. Eleven had positive modified Valsalva tests. Ten were found subsequently to have posterior fossa pathologies (secondary cough headache: 8 Chiari Type 1 malformations, 2 posterior fossa meningiomas). The cough headache was relieved following surgery in all cases. One patient with a positive Valsalva test had an apparently normal brain MRI but measurements of hindbrain and posterior fossa dimensions were consistent with ‘posterior fossa crowdedness’ [2]. The remaining 5 patients had negative (4 patients) or equivocal (1 patient) Valsalva tests and normal MRI scans (primary cough headache).

They suggest that primary and secondary cough headache result from a different pathogenetic mechanism. Primary cough headache appears to be caused by a congestion of the orbital venous plexus in the presence of jugular venous incompetence and a reduced threshold for trigeminal sensory activation. Secondary cough headache results from a transient increase in intracranial CSF pressure during exertion in the presence of obstruction to normal cerebrospinal fluid dynamics”.

They report a patient (case n° 14) who underwent cervical manipulation by a chiropractic for chronic neck pain caused by whiplash sustained in a road traffic accident. Three days later, she developed scalp formication with some mild background headache but in addition, very severe cough headache, provoked in addition by bending over, sneezing and straining at stool. Brain MRI, angiogram and venogram MRI were normal. However, skull and hindbrain measurements showed that this patient fulfilled the criteria for ‘posterior fossa crowdedness’. On follow up the cough headache resolved spontaneously by 6 months.

I would ask the Author if the hypothesis of spontaneous intracranial hypotension (SIH) syndrome caused by cervical manipulation had been considered in the patient n° 14. SIH is characterized by orthostatic headache, low CSF pressure and diffuse pachymeningeal enhancement on brain MRI [3]. Sometimes the brain MRI shows also brain downward displacement, a picture similar to the posterior fossa crowdedness. SIH generally results from spinal spontaneous CSF leakage, sometimes associated with underlying connective tissue disorders [4]. Treatment is usually conservative. Sometimes an autologous epidural blood patch (EBP) may be necessary [5].

## Correspondence/Findings

Cases of SIH caused by cervical manipulation are described in the literature [6]. Even in our series of about 200 cases we found three cases of patients with SIH caused by cervical manipulation. Patients with SIH who had only cough headache without orthostatic headache have also reported [7]. Furthermore in our series of 182 patients with SIH treated with EBP, we found that after the disappearance of orthostatic headache, about 16 hours after treatment, in about 70% of patients remaining cough headache and in addition headache provoked by Valsalva manoeuvre (bending over, sneezing and straining) for several months. Therefore to rule out that the patient n° 14 was suffering from the SIH she would have to perform brain MRI with gadolinium to detect the possible presence of diffuse pachymeningeal enhancement, indirect pathognomonic sign of SIH. This neuroradiological examination was not reported in the article. Finally, the patient described above would have to perform a follow-up brain MRI to assess the possible disappearance of the posterior fossa crowdedness, as expected in a case of SIH.

## Competing interests

The authors declare that they have no competing interests.
